# Characterization of Okra Seed Protein/Rutin Covalent Complex and Its Application in Nanoemulsions

**DOI:** 10.3390/foods14101672

**Published:** 2025-05-09

**Authors:** Chengyun He, Lu Bai, Yingxuan Zhou, Benguo Liu, Sheng Geng

**Affiliations:** School of Food Science, Henan Institute of Science and Technology, Xinxiang 453003, China; hnkjxyhchy@163.com (C.H.); bailu@stu.hist.edu.cn (L.B.); zyx3819105@163.com (Y.Z.); liubenguo@hist.edu.cn (B.L.)

**Keywords:** okra seed protein, rutin, covalent complex, nanoemulsion

## Abstract

A covalent complex of okra seed protein (OSP) and rutin was prepared using the alkali-induced method and characterized. Its application in nanoemulsions was also evaluated. Multi-spectral analysis confirmed the formation of the covalent complex, with OSP as the main body. With an increasing rutin dosage during the preparation process, the amount of rutin in the complex progressively ascended, and the α-helix structure and surface hydrophobicity of the complex gradually declined. The complex exhibited remarkable ABTS radical scavenging capacity and reducing power, which were proportional to the total phenolic content. The OSP/rutin complex could be utilized for the fabrication of O/W nanoemulsions, which remained stable in terms of droplet size and appearance after 28 days of storage at both 4 °C and 25 °C. Furthermore, lipid oxidation in the nanoemulsion stabilized by the OSP/rutin covalent complex could be effectively inhibited, and the emulsion could enhance the UV irradiation resistance of lutein loaded in the oil phase. Our results can provide a reference for the development of protein–polyphenol covalent complexes.

## 1. Introduction

In foods, the interaction between polyphenols and proteins is inevitable, leading to alterations in their structures, functions, and physicochemical properties, which, in turn, affect the flavor, color, and nutritional value of the food. The non-covalent binding of polyphenols to proteins primarily occurs through weak interactions, such as the van der Waals force, electrostatic interaction, hydrogen bonding, and hydrophobic force, which are reversible and sensitive to environmental changes [1]. The covalent binding of proteins and polyphenols is irreversible, resulting in the formation of structurally stable covalent complexes [2]. The covalent binding of polyphenols to proteins is unavoidable in food processing. In the most common alkali-induced reactions, polyphenols are easily oxidized under alkaline and oxygen conditions to the corresponding quinones, which further react with nucleophilic amino acid residues in the protein chain to form C-S or C-N bonds [3]. The covalent combination of the two can also have various effects on food quality. The bioavailability of polyphenols and the digestibility of proteins are often reduced as a result of their covalent association [4]. The stability of specific products, such as beer and milk tea, can also be compromised as a result [5]. However, the covalent binding of polyphenols to proteins under specific conditions can significantly enhance the emulsifying and antioxidant properties of proteins while reducing their allergenicity [6,7]. Li et al. [8] utilized polyphenols (EGCG and chlorogenic acid) to covalently bind with soybean 11S globulin, thereby altering the protein structure and significantly reducing its allergenicity. Ballon et al. [9] covalently combined lesser mealworm larvae protein with polyphenols to develop new antioxidant emulsifiers and prepare stable oil-in-water (O/W) emulsions.

Okra (*Abelmoschus esculentus* L.) seed protein (OSP) is a high-quality plant protein from okra seeds, being rich in various essential amino acids for the human body. It can serve as a protein supplement for cereal foods [10], emerging as a novel source of plant protein. It has been reported that OSP possesses a diverse array of amino acids and potential antioxidant activity [11]. Ijarotimi et al. [12] found that OSP had inhibitory effects on α-amylase, lipase, α-glucosidase, and ACE enzyme and could be applied in functional foods for anti-diabetes and anti-hypertension. There are few studies on the interaction between OSP and polyphenols. Our team previously investigated the non-covalent binding between OSP and 10 flavonoids and explained their binding behavior by establishing a 3D-QSAR model [13]. However, the covalent binding of OSP to polyphenols has not been reported. A nanoemulsion is a kinetically stable system formed by an aqueous phase, an oil phase, and surfactants, with particle sizes ranging from 20 to 500 nm, exhibiting characteristics such as transparency/semi-transparency and low viscosity. In the food industry, nanoemulsions are primarily employed for functional ingredient delivery. OSP exhibited superior emulsifying performance compared to soy protein isolate (SPI), effectively stabilizing nanoemulsions through its enhanced interfacial activity [14]. However, the stability of OSP emulsions was significantly influenced by environmental factors, particularly the pH. Therefore, there is still room for improvement in the storage stability of its emulsion and its resistance to lipid oxidation. Constructing covalent complexes of OSP with polyphenols and developing corresponding nanoemulsions appears to be a viable strategy to improve the functional properties of emulsions.

Rutin is a flavonoid widely distributed in plants, possessing many bioactivities such as antioxidant, antiviral, anti-inflammatory, and cardiovascular/cerebrovascular disease-inhibiting activities [15]. It is widely used in medicine, food, and cosmetics. However, the poor aqueous solubility and low stability of rutin significantly reduce its bioavailability, greatly limiting its application [16]. Li et al. [17] covalently conjugated rutin with buckwheat protein, which significantly enhanced its hydrophilicity and improved the protein’s foaming properties, stability, and antioxidant capacity. In this study, a covalent OSP/rutin complex was prepared via the alkali-induced method. The structural and physicochemical characteristics of the complex were investigated, and its nanoemulsion was also constructed and evaluated. The findings can facilitate the application of protein–polyphenol covalent complexes and the progression of nanoemulsion technology.

## 2. Materials and Methods

### 2.1. Materials and Chemicals

Okra seeds were provided by Henan Feifan Food Co., LTD (Hebi, China). Rutin and 2,2′-Azino-bis(3-ethylbenzothiazoline-6-sulfonic acid) diammonium salt (ABTS) were both bought from Aladdin (Shanghai, China). Medium-chain triglycerides (MCTs) and 8-Anilino-1-naphthalenesulfonic acid (ANS) were purchased from Yuanye Biotechnology Co. Ltd. (Shanghai, China). Sunflower seed oil was the product of Fulinmen Food Co., Ltd. (Shanghai, China). All other chemicals were of analytical grade.

### 2.2. Extraction of OSP

OSP was prepared based on our previous report [14]. The okra seeds were subjected to pulverization followed by defatting with n-hexane. Defatted okra seed powder was mixed well with distilled water (1:20, *w*/*v*), adjusted to pH 9.0, stirred for 2 h, and then centrifuged for 20 min. The supernatant was collected, and the pH of the supernatant was adjusted to 4.5 with HCl and centrifuged again for 15 min. The precipitate was collected and washed three times, and the pH was adjusted to 7.0. The precipitation was redissolved in distilled water and placed in a 3500 Da dialysis bag for 48 h, and the water was changed every 6 h. After vacuum freeze-drying, OSP could be obtained. The protein content of OSP was determined as 89.03% using the Kjeldahl method.

### 2.3. Preparation of OSP/Rutin Covalent Complex

Covalent complexes of OSP and rutin were prepared via alkali treatment [8]. The OSP was dissolved in 200 mL of distilled water, adjusted to pH 9.0, stirred magnetically for 2 h, and stored overnight at 4 °C. Sodium azide at a concentration of 0.01% was also added to inhibit microbial growth. The following day, 200 mL of rutin solutions at different concentrations (0.02 wt%, 0.05 wt%, and 0.1 wt%) were prepared. Under magnetic stirring, the OSP solution was mixed with the rutin solutions and then stirred overnight at a constant speed of 120 rpm. The pH of the mixture was always kept at 9.0. Finally, the mixture was dialyzed in a 3500 Da dialysis bag for 48 h. The covalent complexes of OSP and rutin were obtained via vacuum freeze-drying, and the complexes were designated as OSP/Rutin-1, OSP/Rutin-2, and OSP/Rutin-3, respectively.

### 2.4. Determination of Ultraviolet Spectrum (UV)

OSP, OSP/Rutin-1, OSP/Rutin-2, and OSP/Rutin-3 aqueous solutions at 0.2 mg/mL and rutin aqueous solution at 0.04 mg/mL were prepared. Distilled water was used as a blank control. Their UV spectra (220–400 nm) were measured using a spectrophotometer with a scanning interval of 1.0 nm.

### 2.5. Measurement of Fourier Transform Infrared Spectra (FT-IR)

The OSP, rutin, and OSP/rutin covalent complex were utilized as analytical samples. Under a drying lamp, they were mixed and ground at a ratio of sample to potassium bromide of 1:100, followed by pellet formation. Spectral scanning was carried out using a FT-IR spectrometer, using potassium bromide as a blank background, setting the resolution to 4 cm^−1^ and the scanning range to 400–4000 cm^−1^.

### 2.6. Determination of Circular Dichroism (CD)

The secondary structure of the samples was analyzed using a J-1500 circular dichroism spectrometer (JASCO, Tokyo, Japan) with a scan rate of 50 nm/min. The scanning range was 190–240 nm, and the optical path of the sample pool was 1 mm. The obtained CD spectral data were uploaded to the website, http://dichroweb.cryst.bbk.ac.uk (accessed on 12 March 2025), where the CONTIN algorithm was employed to calculate the relative content of secondary structures [18,19,20].

### 2.7. Measurement of Endogenous Fluorescence (FS)

OSP and OSP/rutin covalent complexes were weighed and dispersed in PBS buffer (10 mmol/L, pH 7.0) and diluted to 1.0 mg/mL. Spectral scanning was performed using an Agilent Cary Eclipse fluorescence spectrophotometer (Santa Clara, CA, USA), using the fluorophore of OSP as a probe [21]. The emission spectrum (300–450 nm) at the excitation wavelength of 280 nm was recorded at a slit width of 5 nm and a scanning speed of 600 nm/min.

### 2.8. Determination of Total Phenolic Content (TPC)

The Folin–Ciocalteu method was used to determine the total phenolic content of the OSP/rutin covalent complex [22]. OSP/Rutin-1, OSP/Rutin-2, and OSP/Rutin-3 covalent complex solutions were prepared at 5 mg/mL, respectively. A total of 0.3 mL of the sample solution was diluted to 10 mL in a 25 mL volumetric flask. Subsequently, 0.5 mL of Folin–Ciocalteu reagent and 5 mL of sodium NaCO_3_ solution (5%, *w*/*v*) were sequentially added to the volumetric flask. The mixture was diluted to 25 mL and kept for 90 min in the dark. Then, its absorbance at 750 nm was measured, with distilled water as the blank control. The total phenolic content in the sample was determined using a rutin standard curve, with the result expressed as milligrams of rutin per gram of sample (mg/g).

### 2.9. Evaluation of Surface Hydrophobicity

Referring to the report of Zhang et al. [23], ANS was employed as a fluorescent probe to investigate the effect of rutin on the surface hydrophobicity of OSP. Aqueous protein solution (0.05–0.30 mg/mL) was prepared with PBS buffer solution (10 mmol/L, pH 7.0). The OSP aqueous solution (4 mL) was mixed with the ANS solution (20 μL, 8 mmol/L) and kept in the dark for 30 min. The fluorescence spectrum of the mixture in the range of 400–700 nm was recorded at an excitation wavelength of 390 nm, with the OSP solution without ANS serving as the blank. The maximum fluorescence intensity was plotted against the protein concentration, with the slope of the resulting linear regression representing the relative surface hydrophobicity index (H_0_).

### 2.10. ABTS Free Radical Scavenging Assay

According to the method of Fogarasi et al. [24], the ABTS radical scavenging capacities of OSP, rutin, and OSP/rutin covalent complexes were determined. Firstly, 100 mL of ABTS (7 mmol/L) solution was prepared, mixed with 1.75 mL of potassium persulfate (2.45 mmol/L), and reacted in the dark for more than 12 h to generate ABTS radicals. Then, the obtained solution was diluted with PBS buffer (0.05 mol/L, pH 7.4) to achieve an absorbance of 0.7 ± 0.01 at 734 nm and subsequently collected as the ABTS test solution. The 1 mL sample solution (OSP, rutin, OSP/Rutin-1, OSP/Rutin-2, and OSP/Rutin-3) with different concentrations (0–15 μg/mL for rutin; 0–1000 μg/mL for OSP and OSP/rutin complexes) were mixed with 2 mL of ABTS test solution and reacted in the dark for 10 min. The absorbance value at 734 nm was measured and recorded as *A*_sample_. The control group consisted of 1 mL of PBS instead of the sample solution, denoted *A*_control_. The ABTS free radical scavenging activity of the sample for each concentration was calculated according to Formula (1):

(1) $$\mathrm{ABTS}\;\mathrm{free}\;\mathrm{radical}\;\mathrm{scavenging}\;\mathrm{activity}=\frac{A_{\mathrm{control}}-A_{\mathrm{sample}}}{A_{\mathrm{control}}}\times 100\%$$

### 2.11. Reducing Power Assay

According to the method of Zhou et al. [25], the sample solutions of different concentrations (0–1.0 μg/mL for rutin; 0–10 μg/mL for OSP and OSP/rutin complexes), potassium ferricyanide solution (1%, *w*/*v*), trichloroacetic acid solution (10%, *w*/*v*), and FeCl_3_ solution (0.1%, *w*/*v*) were prepared with PBS buffer (0.2 mol/L, pH 6.6). Then, 0.5 mL of sample solution, 2.5 mL of PBS, and 2.5 mL of potassium ferricyanide solution were mixed in a 10 mL centrifuge tube and incubated in water at 50 °C for 20 min. After the reaction, they were cooled rapidly. Subsequently, 2.5 mL of trichloroacetic acid was added and centrifuged at 3000× *g* for 10 min. Finally, 2.5 mL of the supernatant, 2.5 mL of distilled water, and 0.5 mL of FeCl_3_ solution were mixed and allowed to react for 10 min. The absorbance of the mixture was then measured at 700 nm, which reflected the reducing power of the sample.

### 2.12. Preparation of Nanoemulsions

In accordance with our previous report [14], the ultrasonic emulsification method was employed to prepare nanoemulsions. Specifically, 0.5 g of the samples (OSP, OSP/Rutin-1, OSP/Rutin-2, and OSP/Rutin-3) was dissolved in 95 mL of distilled water with 0.01% sodium azide. It was stirred for 2 h and mixed with 5 mL of MCTs. The mixture was subjected to high-shear processing (15,000 rpm, 2 min) using an IKA T18 high-speed shear homogenizer (Staufen, Germany), resulting in a coarse emulsion. Then, the nanoemulsion was prepared using a Scientz JY99-IIDN ultrasonic homogenizer (Ningbo, China). The ultrasonic power was set to 600 W, the ultrasonic time was set to 340 s, the ultrasonic working time and the intermittent time were set to 2 s, and the temperature was controlled using an ice bath to ensure it remained below 25 °C. The resulting O/W nanoemulsions were named OSP-Emulsion, OSP/Rutin-1-Emulsion, OSP/Rutin-2-Emulsion, and OSP/Rutin-3-Emulsion, respectively.

### 2.13. Storage Stability Experiment

Four emulsions were prepared according to Section 2.12. The resulting emulsions were stored at 4 °C and 25 °C for 28 days, during which the droplet size and appearance changes were monitored periodically. The droplet size of the emulsion was measured using a Nano-ZS nanoparticle size analyzer (Malvern Instruments, Worcestershire, UK) after equilibration for 60 s at 25 °C. To mitigate the effects of multiple light scattering, the emulsion sample was diluted 1000-fold with distilled water prior to analysis.

### 2.14. Evaluation of Anti-Lipid Oxidation Capacity

Sunflower seed oil was used as the oil phase to prepare the nanoemulsions according to Section 2.12. The antioxidant capacity against lipid oxidation of the four emulsions was evaluated using the method described by Li et al. [26]. The obtained nanoemulsion was stored at 50 °C. Every five days, 200 μL of the emulsion was withdrawn and placed in a 10 mL centrifuge tube, followed by the addition of 1.5 mL of iso-octane/2-propanol (3:1, *v*/*v*). The sample was vortexed three times for 10 s each and then centrifuged at 2000× *g* for 5 min using a high-speed centrifuge. After standing for 3 min, 200 μL of the supernatant was taken into a centrifuge tube, and 2.8 mL of methanol/1-butanol (2:1, *v*/*v*), 15 μL of 3.94 mol/L ammonium cyanide sulfate, and 15 μL of ferrous solution (a mixture of 0.132 mol/L barium chloride and 0.144 mol/L ferrous sulfate) were added. The mixture was swirled for 30 s and left for 20 min. The absorbance at 510 nm was recorded, and the peroxide concentration was determined based on the hydrogen peroxide standard curve.

### 2.15. Determination of Protective Effect of Lutein

According to the method of Wang et al. [27], lutein was dissolved in MCTs until its concentration reached 2.0 mg/mL, which was used as the oil phase. Then, the lutein nanoemulsions were prepared according to Section 2.12. The nanoemulsions were incubated at 30 °C and exposed to a 6W UV lamp placed 20 cm away. The degradation of lutein was monitored. Briefly, 1 mL of the nanoemulsion was taken and mixed with 6 mL of hexane/ethanol (2:1, *v*/*v*) and 1 mL of distilled water. After vortexing for 30 s, the nanoemulsion was centrifuged (2000× *g*, 6 min) in a high-speed centrifuge and left for 10 min in the dark. The absorbance of the supernatant at 450 nm was measured to determine the lutein content in the nanoemulsion based on the lutein standard curve. MCTs containing 2.0 mg/mL of lutein were used as a control. The lutein retention rate (R) was calculated using Formula (2):

(2) $$R=\frac{C_{t}}{C_{0}}\times 100\%$$

where *C*_0_ and *C_t_* are the lutein contents of the lutein nanoemulsion at time = 0 and time = *t*, respectively.

### 2.16. Statistical Analysis

Each measurement was performed in triplicate, and the result was expressed as the mean ± standard deviation. The experimental data were subjected to variance analysis, correlation analysis, and Duncan’s multiple comparison using the statistical software SPSS 18. Data visualization was performed using Origin 2021 software.

## 3. Results and Discussion

### 3.1. UV Analysis

**Figure 1:**
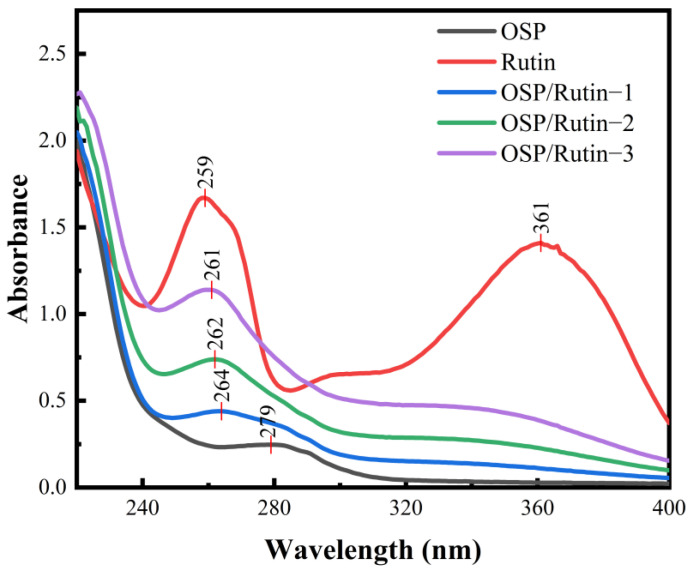
Ultraviolet–visible spectra of OSP, rutin, and the OSP/rutin covalent complexes.

UV spectra can characterize the conjugated structure of unsaturated compounds and aromatic compounds [28], which can reflect the structural changes after covalent binding of proteins and flavonoids. Figure 1 exhibited the UV spectra of OSP, rutin, and OSP/rutin complexes. OSP had an absorption maximum at 279 nm because the protein contained aromatic amino acids such as phenylalanine, tyrosine, and tryptophan. Rutin, a flavonol compound, exhibited two characteristic absorption peaks at 259 nm and 361 nm, respectively. After the covalent reaction, the UV absorption of the complexes gradually ascended with increasing rutin usage. Shi et al. also reported a similar experimental phenomenon, which is due to the covalent binding of OSP and rutin causing the structure of the protein to unfold [29]. Moreover, compared to OSP, the OSP/rutin covalent complexes exhibited a significant blue shift, demonstrating the characteristic UV spectral peaks of rutin. This indicated that the addition of rutin resulted in conformational changes in OSP, altering the microenvironment of the side-chain amino acid residues (tryptophan and tyrosine). It was preliminarily concluded that the formation of the OSP/rutin conjugate was directly proportional to the amount of rutin, indicating a correlation between the degree of covalency and the quantity of rutin.

### 3.2. FT-IR Analysis

**Figure 2:**
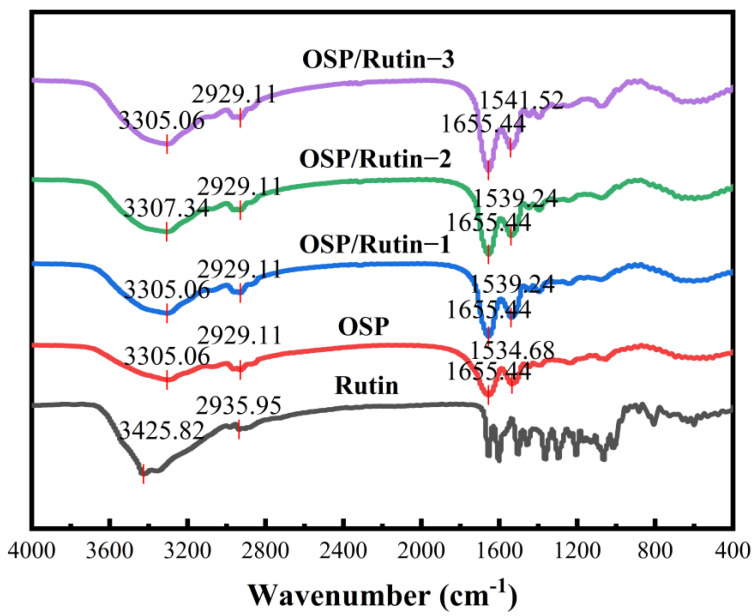
FT-IR spectra of OSP, rutin, and the OSP/rutin covalent complexes.

FT-IR spectroscopy has found extensive application in the analysis of protein conformational changes, protein–ligand interactions, and quality monitoring in bio-pharmaceuticals. Figure 2 presents the FT-IR spectra of OSP, rutin, and OSP/rutin covalent complexes. OSP exhibited prominent absorption peaks at 3305.06, 2929.11, 1655.44, and 1534.68 cm^−1^, corresponding to the amide A band (stretching vibrations of hydrogen bonds and N-H bonds), the amide I band (stretching vibrations of C=O bonds), and the amide II band (stretching of C-N and bending of N-H bonds), respectively [30]. Rutin had a strong absorption peak between 3200 and 3600 cm^−1^, with a relatively broad peak shape, corresponding to the stretching vibrations of phenolic hydroxyl groups. Simultaneously, a sharp absorption peak was observed at 2935.95 cm^−1^, which was due to the stretching vibrations of C-H bonds. It was evident that the FT-IR spectra of the complexes primarily exhibited the spectral characteristics of OSP. With the increase in rutin concentration, the absorption peaks of each waveband intensified, and the amide II band exhibited a blue shift of 4.56–6.84 cm^−1^. Han et al. [31] also found a similar experimental phenomenon, indicating that the addition of rutin caused tensile vibration of OSP, which changed the secondary structure of OSP.

### 3.3. CD Analysis

**Figure 3:**
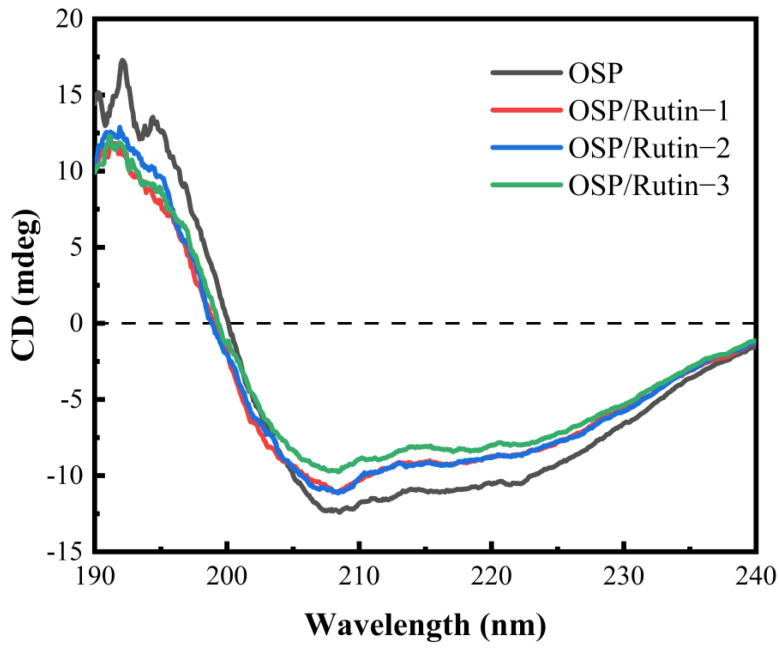
Circular dichrogram plot of OSP and the OSP/rutin covalent complexes.

CD is frequently employed to characterize changes in the secondary structure of proteins upon interaction with ligands. The far-UV region (190–240 nm) can reflect the conformational change of the protein skeleton. As shown in Figure 3, OSP exhibited negative peaks at 208 nm and 222 nm, characteristic of an α-helix structure, and concurrently displayed a peak characteristic of a β-sheet structure at 218 nm [32], indicating that the secondary structure of OSP comprised relatively high proportions of both α-helices and β-sheets. However, after covalent conjugation of OSP with rutin, the peak intensities at 208 nm and 222 nm were attenuated, and shifts of varying degrees occurred. This is because upon the formation of covalent complexes between OSP and rutin, rutin induced conformational changes in OSP (Table 1). The addition of rutin caused the unwinding of the α-helix structure of OSP, leading to a transition towards other structural conformations. Usually, the content of α-helices correlates with the rigidity of the protein. Such transformations led to a decrease in the proportion of ordered structures within OSP, while the proportion of disordered structures ascended. Consequently, the overall structure became more loosely organized and had enhanced flexibility, thereby facilitating interactions with small molecules [33]. Liu et al. [34] reported that covalent binding of caffeic acid and ovalbumin also resulted in a decline in α-helices and an increase in random coils.

### 3.4. FS Analysis

**Figure 4:**
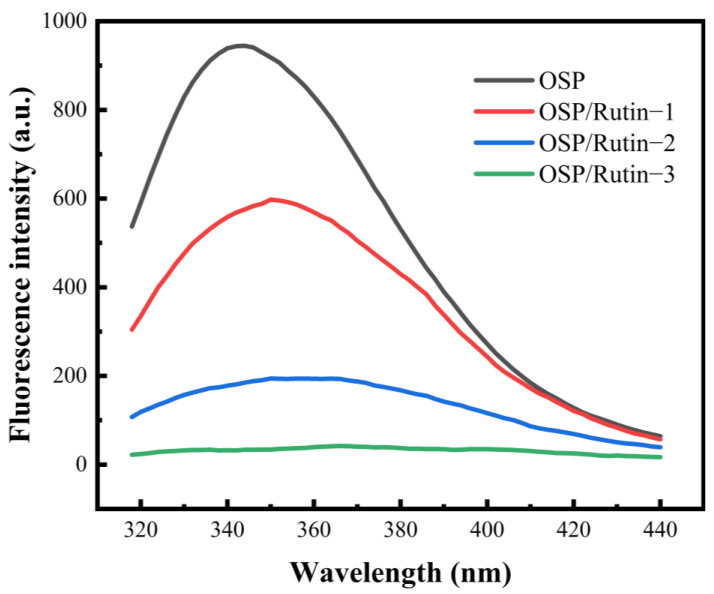
Intrinsic fluorescence spectra of OSP and the OSP/rutin covalent complexes.

Intrinsic FS analysis of proteins and polyphenol–protein covalent complexes is an important technical means to explore their structures and interactions. For proteins containing fluorescent chromophores, changes in their microenvironment can be detected by exciting the aromatic amino acid residues (tryptophan, tyrosine, and phenylalanine) within them [35]. In Figure 4, the FS intensity of the covalent complex declined significantly with the increasing amount of rutin added, in comparison to OSP. Furthermore, the fluorescence spectra of OSP/Rutin-1, OSP/Rutin-2, and OSP/Rutin-3 exhibited varying degrees of redshift ranging from 6 to 22 nm. The report of Cheng et al. [36] also observed similar experimental phenomena. It confirmed that under alkaline induction conditions, multiple active groups of rutin, such as phenolic hydroxyl groups, interacted with aromatic amino acids on OSP, and this interaction altered the microenvironment of amino acid residues, leading to the exposure of tryptophan and tyrosine residues, which were originally situated within hydrophobic regions, to a more hydrophilic environment. The increased polarity of the surrounding microenvironment caused a redshift in the fluorescence spectrum.

### 3.5. Total Phenolic Content

To further substantiate the covalent linkage between OSP and rutin, the total phenolic content of the OSP/rutin covalent complex was measured using the Folin–Ciocalteu method. As can be seen in Table 2, the total phenol contents of OSP/Rutin-1, OSP/Rutin-2, and OSP/Rutin-3 were 64.81 ± 1.51, 93.40 ± 0.42, and 123.92 ± 1.38 mg/g, respectively. The total phenolic content of the covalent complex ascended with the increase in rutin addition. Rutin contains multiple phenolic hydroxyl groups. These phenolic hydroxyl groups had certain chemical reactivity and provided many reaction sites for covalent binding with OSP. OSP consisted of amino acid residues and had a variety of reactive groups, such as amino groups, mercapto groups, carboxyl groups, etc., which could participate in the reaction with rutin. Under aerobic and alkaline conditions, the phenolic hydroxyl groups of rutin were easily oxidized to form ortho-quinone or semiquinone intermediates, which further reacted with amino acid residues of OSP to form the OSP/rutin covalent complex [37].

### 3.6. Surface Hydrophobicity Analysis

**Figure 5:**
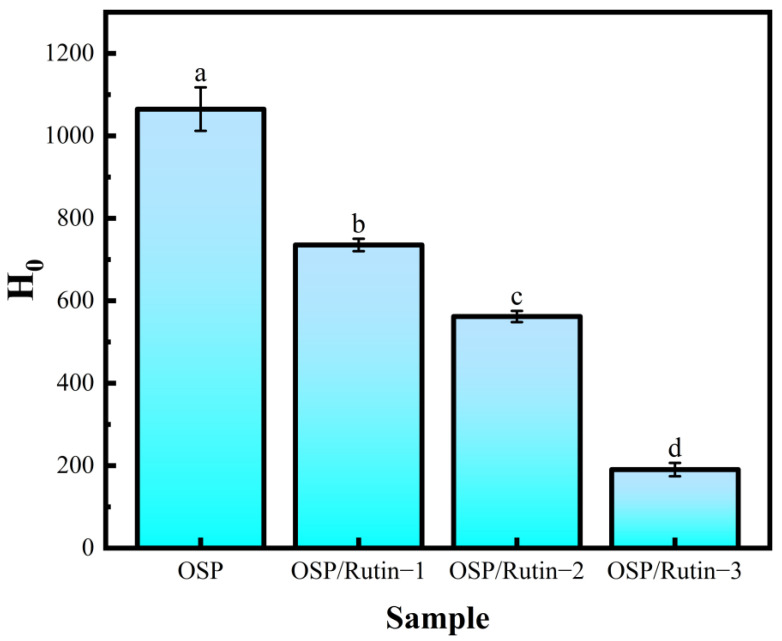
Surface hydrophobicity of OSP and the OSP/rutin covalent complexes (different letters in the figure indicate statistically significant differences, *p* < 0.05).

For the surface hydrophobicity assay, H_0_ reflects the quantity of hydrophobic groups in contact with the environment, and alterations in H_0_ can further influence the functional properties of proteins, such as emulsifying capacity and solubility [38]. Wang et al. reported that the emulsifying properties of soy protein varied with changes in surface hydrophobicity [39]. In Figure 5, compared to OSP, the H_0_ value of the OSP/rutin covalent complex was significantly reduced. Notably, the H_0_ value for OSP/Rutin-3 reached the lowest level at 190.43 ± 16.32. This indicated that covalent bonding with the hydrophilic groups of rutin (such as phenolic hydroxyl groups) led to the disruption of hydrophobic amino acids within the OSP molecule, thereby increasing its hydrophilicity. This conclusion was also validated in the fluorescence spectra of both OSP and the OSP/rutin covalent complex. Sun et al. found that the stability of the emulsion could be enhanced by adjusting the interfacial properties (surface hydrophobicity, etc.) of the complex particles [40].

### 3.7. ABTS Free Radical Scavenging Ability

**Figure 6:**
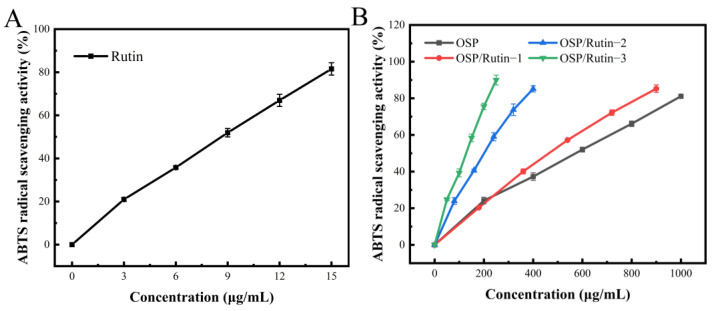
ABTS radical scavenging ability of rutin (**A**), OSP, and the OSP/Rutin covalent complexes (**B**).

The ABTS test is a commonly employed method for evaluating antioxidant capacity. Upon oxidation, ABTS generates a stable blue-green cation radical, ABTS^+^, which exhibits maximum absorption at a 734 nm [41]. The antioxidant activity of the samples was expressed in terms of ABTS^+^ radical scavenging capacity, wherein a higher scavenging rate indicates a stronger antioxidant ability. In Figure 6A,B, the ABTS radical scavenging capacities of OSP, rutin, and the OSP/rutin covalent complex ascended with an increasing concentration, being proportional to the total phenolic content. The IC_50_ of the OSP/rutin covalent complex was significantly lower than that of OSP alone (Table 3). The antioxidant capacity of the covalent complex increased by 15.54%, 64.26%, and 78.14%, respectively, suggesting that covalent binding with rutin enhanced the antioxidant activity of OSP. This was primarily attributed to the introduction of phenolic hydroxyl groups from the rutin molecule into the OSP molecule following covalent reactions, wherein hydroxyl groups make a significant contribution to the antioxidant capacity of the substance [42].

### 3.8. Reducing Power

**Figure 7:**
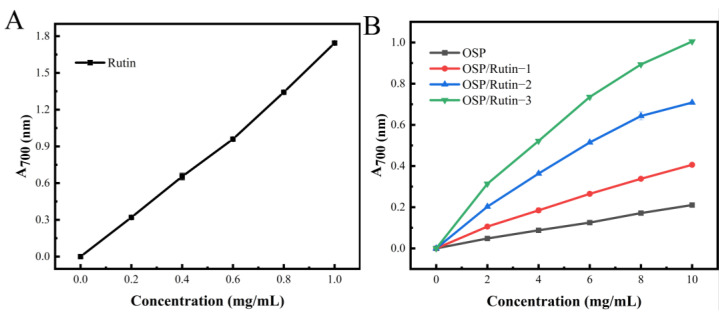
Reducing power of rutin (**A**), OSP, and the OSP/rutin covalent complexes (**B**).

The ferric reducing ability refers to the capacity of the sample to reduce Fe^3+^ to Fe^2+^, which is an important indicator reflecting the antioxidant properties of the sample. The antioxidant is capable of reducing Fe^3+^ in potassium ferricyanide (K_3_Fe(CN)_6_) to Fe^2+^, which subsequently reacts with FeCl_3_ to form Prussian blue (Fe_4_[Fe(CN)_6_]_3_), which has a characteristic absorption peak at 700 nm, and its absorbance value is positively correlated with the reducing power [43]. The reducing powers of OSP, rutin, and the OSP/rutin covalent complex are illustrated in Figure 7A,B. Rutin exhibited the strongest reducing power, followed by the OSP/rutin covalent complex. When the sample concentration was 10.0 mg/mL, the reducing power of OSP/Rutin-1, OSP/Rutin-2, and OSP/Rutin-3 was 1.93 times, 3.37 times, and 4.78 times that of natural OSP, which was proportional to the total phenolic content. The results suggested that the higher the combining amount of rutin, the higher the number of phenolic hydroxyl groups in the OSP/rutin complex and the stronger the reducing power of the samples. Similar to our study, Yi et al. [44] found that the reducing power of the catechin–α-lactalbumin covalent complex was significantly higher than that of the native protein. These studies have shown that covalent modification of proteins with polyphenols can significantly improve the antioxidant capacity of proteins.

### 3.9. Storage Stability Analysis

**Figure 8:**
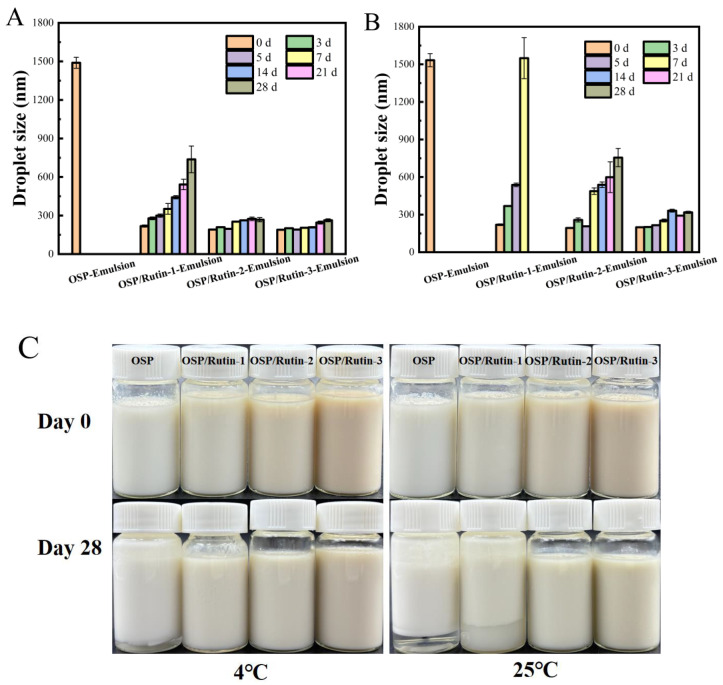
The variation in droplet size of nanoemulsions stored at 4 °C (**A**) and 25 °C (**B**), along with changes in the emulsion appearance (**C**).

The physical stability of emulsions during manufacturing, transportation, storage, and usage is a critical aspect of their practical application. External affecting factors include mechanical shear forces and temperature during manufacturing, vibrations/temperature fluctuations during transportation, light exposure/microbial contamination/oxygen exposure during storage, and environmental pH variations/mechanical disturbances/temperature changes during usage. Temperature variations can influence the stability of emulsions through multiple mechanisms. The droplet size reflects the degree of aggregation of emulsion droplets, which significantly influences various aspects of the emulsion, including its properties, appearance, and application performance. The smaller the droplet size, the higher the stability of the emulsion, and prolonged storage is less likely to result in sedimentation and phase separation [45]. Polyphenols can significantly alter the secondary structure of proteins through covalent binding, leading to a decrease in sulfhydryl groups and tyrosine residues, promoting the exposure of molecular hydrophobic regions, and enhancing surface activity. This structural modification renders the composite more prone to adsorb at the oil–water interface, thereby reducing interfacial tension. Furthermore, the incorporation of polyphenols increases the hydroxyl group density on molecular surfaces, forming a denser interfacial film through hydrogen bonding and electrostatic repulsion, which effectively inhibits droplet coalescence. This study systematically investigated the changes in droplet size and appearance of four emulsions stored at 4 °C and 25 °C over a period of 28 days (Figure 8). The droplet size of the freshly prepared OSP-Emulsion was approximately 1500 nm, whereas the emulsions prepared from the OSP/rutin covalent complex exhibited droplet sizes below 220 nm, characteristic of typical nanoemulsions [46]. This result suggested that the increased hydrophilicity of the OSP/rutin covalent complex could effectively reduce the interfacial tension between the two phases at the oil–water interface of the emulsion, promoting the formation of smaller and more uniform droplets to form a stable nanoemulsion. With the extension of storage time, the droplet sizes of the emulsions all increased to varying degrees. Among them, the OSP-Emulsion was the most unstable, with the largest change in droplet size. After being stored for three days at 4 °C and 25 °C, a phenomenon of oil–water separation was observed in both conditions. At the same time, nanoemulsions formed by covalent complexes had smaller particle sizes and a more stable emulsion appearance. After 28 days of storage, the droplet sizes of OSP/Rutin-3-Emulsion were 263 nm (4 °C) and 318 nm (25 °C) respectively, both remaining below 320 nm. Compared to 25 °C, the variation in the droplet size of the emulsion at 4 °C was relatively mild. This was attributed to the fact that higher temperatures accelerated molecular motion, thereby increasing the collision frequency among droplets within the emulsion. If the repulsive forces between droplets were insufficient to overcome the attractive forces during collisions, it could facilitate droplet coalescence, leading to an increase in the droplet size [47].

### 3.10. Anti-Lipid Oxidation Capacity

**Figure 9:**
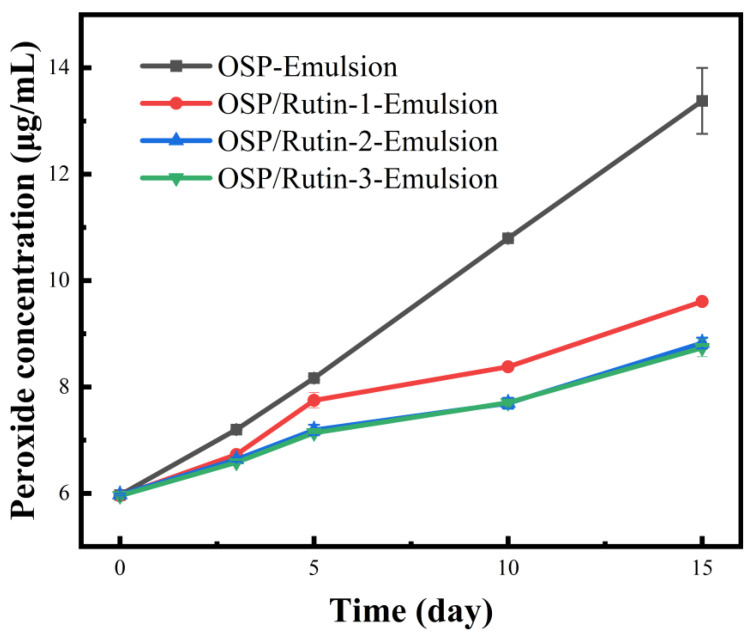
Changes in peroxide content of nanoemulsions during storage.

Nanoemulsions possess a large specific surface area, allowing for extensive contact between the lipids in the oil droplets and oxidants at the oil–water interface, thereby facilitating oxidation [48]. Furthermore, sunflower seed oil contains high levels of oleic and linoleic acids, which render the nanoemulsions more susceptible to oxidation. To evaluate their anti-lipid oxidation capability, the emulsions were prepared using sunflower oil, and the changes in peroxide content of each emulsion were examined over a 15-day storage period at 50 °C. As illustrated in Figure 9, the peroxide content ascended over time with extended storage. The most rapid increase in peroxide content was observed in OSP-Emulsion, followed by OSP/Rutin-1-Emulsion. The increases in OSP/Rutin-2-Emulsion and OSP/Rutin-3-Emulsion were relatively similar, which may be attributed to the strong antioxidant properties of rutin. When its concentration exceeds a certain threshold, the antioxidant effect remains stable over a specific period. Geng et al. [49] also reported similar findings in the construction of Pickering nanoemulsions, noting that when dihydromyricetin exceeds a certain concentration, there is no significant difference in the antioxidant effects among the emulsions. It could be inferred that the nanoemulsion constructed from the covalent complex of OSP/rutin effectively inhibited the formation of peroxides, thereby maintaining the quality of the emulsion and extending its shelf life. This is because the strong antioxidant activity of rutin could directly inhibit lipid oxidation, while the OSP/rutin covalent complexes also enhanced the interfacial physical barrier to block the diffusion of oxidizing agents.

### 3.11. Lutein Stability Analysis

**Figure 10:**
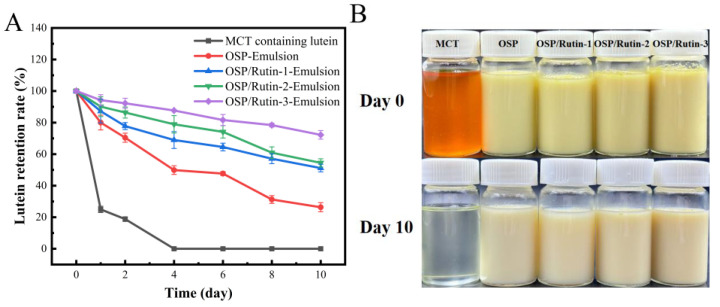
Lutein retention rate (**A**) and appearance (**B**) of nanoemulsions under UV irradiation.

Lutein is a naturally occurring fat-soluble bioactive ingredient with strong antioxidant activity and can effectively resist ultraviolet radiation and prevent diseases such as senile macula and cataracts [50]. However, lutein is characterized by instability, exhibiting sensitivity to changes in external environmental factors such as the pH, ionic strength, temperature, light, and oxygen. This instability limits its application in foods. Frede et al. found that the construction of lutein-loaded emulsions can effectively prevent the oxidation of lutein and improve the stability of lutein. Therefore, this study investigated the capability of the emulsions developed by the OSP/rutin covalent complexes to protect lutein from UV irradiation [51]. In Figure 10A, all emulsions exhibited superior lutein protective capacity compared to the control group MCT. On the fourth day of UV irradiation at 30 °C, the lutein in MCT decreased to 0. At this point, the lutein retention rates of OSP-Emulsion, OSP/Rutin-1-Emulsion, OSP/Rutin-2-Emulsion, and OSP/Rutin-3-Emulsion, were 49.89%, 68.94%, 78.94%, and 87.67%, respectively. It was obvious that the lutein protection effect of nanoemulsions constructed covalent complexes was significantly superior to that of OSP-Emulsion, which was attributed to the antioxidant capacity and UV absorption of rutin. In particular, after 10 days of UV irradiation, the retention rate of lutein in the nanoemulsion constructed the covalent complex remained above 50%. The protective effect of the nanoemulsion prepared with the OSP/rutin covalent complex on lutein was better than that prepared using a dihydromyricetin/α-lactalbumin covalent complex [43]. Based on the appearance of the emulsions (Figure 10B), after 10 days of UV irradiation at 30 °C, the lutein in the MCTs had completely disappeared, and all the emulsions had undergone discoloration, with OSP-Emulsion exhibiting the most pronounced fading.

## 4. Conclusions

This study utilized the alkali-induced method to prepare OSP/rutin covalent complexes. The spectral analysis indicated that OSP underwent changes in its structure and microenvironment. The covalent binding with rutin significantly enhanced the antioxidant activity and hydrophilicity of OSP. Moreover, the addition amount of rutin was positively correlated with the total phenolic content, hydrophilicity, and antioxidant activity of the OSP/rutin covalent complex. The droplet size of the emulsions developed by the OSP/rutin covalent complexes were all lower than 220 nm. These emulsions showed higher storage stability, anti-oxidation ability, and lutein protection effects, which were superior to the emulsion stabilized by OSP. Despite the promising results, the study identified a potential issue regarding the long-term stability of the nanoemulsions, particularly under higher temperature conditions (25 °C). Only OSP/Rutin-3-Emulsion remained stable after 28 days of storage, while the droplet size of the other emulsions increased significantly with time, indicating the need for further optimization to improve long-term storage stability.

## Figures and Tables

**Table 1:** Secondary structure contents of OSP and OSP/rutin covalent complexes.

Samples	α-Helix (%)	β-Sheet (%)	β-Turn (%)	Random (%)
OSP	17.9 ^a^	30.9 ^c^	12.7 ^b^	38.5 ^c^
OSP/Rutin-1	15.9 ^b^	32.1 ^b^	12.9 ^ab^	39.1 ^b^
OSP/Rutin-2	15.4 ^c^	32.2 ^b^	13.0 ^a^	39.3 ^a^
OSP/Rutin-3	15.0 ^d^	33.0 ^a^	12.9 ^ab^	39.2 ^ab^

Different superscript letters indicate statistically significant differences within the same column (*p* < 0.05).

**Table 2:** Total phenolic content of OSP and OSP/rutin covalent complexes.

Rutin Structure	Sample	Total Phenol Content (mg/g)
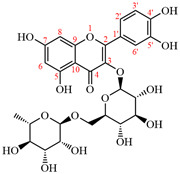	OSP/Rutin-1	64.81 ± 1.51 ^c^
	OSP/Rutin-2	93.40 ± 0.42 ^b^
	OSP/Rutin-3	123.92 ± 1.38 ^a^

Different superscript letters indicate statistically significant differences within the same column (*p* < 0.05).

**Table 3:** ABTS cation scavenging assays for OSP, rutin, and OSP/rutin covalent complexes.

Sample	IC_50_ (μg/mL)
Rutin	7.67 ± 0.15 ^e^
OSP	497.24 ± 5.30 ^a^
OSP/Rutin-1	419.96 ± 5.99 ^b^
OSP/Rutin-2	177.72 ± 5.40 ^c^
OSP/Rutin-3	108.71 ± 0.81 ^d^

Different superscript letters indicate statistically significant differences within the same column (*p* < 0.05).

## Data Availability

The original contributions presented in the study are included in the article; further inquiries can be directed to the corresponding author.
